# Dose determination of VV116 in COVID-19 patients with severe liver dysfunction: a case report

**DOI:** 10.3389/fmed.2025.1541235

**Published:** 2025-02-25

**Authors:** Jing Yang, Wenwen Jiang, Jianqing Deng, Min Liu, Ya Xue, Jizhang Bao, Tingting Jia, Qi Hu, Lichao Zhang

**Affiliations:** ^1^Department of Pharmacy, Shanghai Municipal Hospital of Traditional Chinese Medicine, Shanghai University of Traditional Chinese Medicine, Shanghai, China; ^2^Department of Hematology, Shanghai Municipal Hospital of Traditional Chinese Medicine, Shanghai University of Traditional Chinese Medicine, Shanghai, China

**Keywords:** COVID-19, VV116, liver dysfunction, dosage adjustment, SARS-CoV-2

## Abstract

VV116 is an oral antiviral drug against SARS-CoV-2, known for its favorable efficacy and safety profile. But its application in patients with severe liver dysfunction has not been evaluated. Here, we report a case in which a patient with aplastic anemia and liver impairment (recovery phase of acute liver failure) was infected with SARS-CoV-2. Based on clinical trials and pharmacokinetic analysis about VV116, we initiated a reduced dose of 300 mg every 12 h on day 1, 200 mg every 12 h on days 2–5 for antiviral therapy. Finally, the patient’s viral load rapidly dropped to an undetected level, and no drug-related adverse effects were observed.

## 1 Introduction

Liver dysfunction is prevalent in COVID-19 patients, affecting approximately 50% of infected individuals (1, 2). This prevalence can be attributed to two main factors. On one hand, liver diseases are widespread, affecting over 300 million people in China alone (3); and liver dysfunction is a risk factor of COVID-19 infection and disease progression (4, 5). On the other hand, SARS-CoV-2 infection itself has certain impairment on liver function (6). However, antiviral drugs suitable for COVID-19 patients with liver dysfunction are limited.

Currently, the treatment regimens for COVID-19 mainly include two classes, neutralizing antibodies hindering viral entry by targeting the Spike protein and small-molecule antivirals suppressing the replication of SARS-CoV-2 by targeting the conserved RNA-dependent RNA polymerase (RdRp) or main protease (Mpro). The usage of neutralizing antibody is limited due to the inconvenient administration route and drug resistance to the emerging SARS-CoV-2 subvariants (such as the XBB lineage) (7, 8). The approved or authorized small-molecule antivirals include nirmatrelvir-ritonavir (Paxlovid), remdesivir, molnupiravir, deuremidevir hydrobromide (VV116), etc. Among these small-molecule drugs, Paxlovid ranks first in reducing mortality and hospitalization, and VV116 ranks first in safety outcomes from a network meta-analysis (9). Paxlovid is a co-packaged combination agent consisting of nirmatrelvir and ritonavir, among which nirmatrelvir is the inhibitor of Mpro and ritonavir is a pharmacologic booster of nirmatrelvir (10). The main concern of Paxlovid in clinic use is the multiple drug-drug interactions, mainly owing to that ritonavir is a strong CYP3A4 inhibitor (11, 12). Additionally, ritonavir has potential hepatotoxicity (13). VV116 is a deuterated remdesivir hydrobromide showing potent anti-SARS-CoV-2 activity by inhibiting RdRp (14, 15). VV116 exhibits no mutagenicity compared with molnupiravir and has fewer drug-drug interactions compared with Paxlovid (16). Moreover, VV116 shows favorable pharmacokinetics properties conferred by its deuteration modification (17).

Here, we present a case of patient with aplastic anemia and severe liver dysfunction who was infected with Omicron XBB.1, treated with VV116, and eventually recovered.

## 2 Case presentation

A 40-year-old male was diagnosed with acute liver failure on January 17, 2024, followed by timely plasma exchange and systemic anti-inflammatory therapy. One week later, the patients developed pancytopenia and aplastic anemia was diagnosed. On February 3, 2024, he was transferred to the department of hematology of our hospital for further treatment. On admission, his clinical symptoms included skin petechiae and ecchymosis, jaundice, anorexia, nausea, fatigue, cough with expectoration, headache and chest pain. The laboratory test data was shown in Table 1. The initial treatment consisted of hepatoprotective therapy and supportive care like blood transfusion.

**TABLE 1:** Laboratory test data of the patient on admission.

Laboratory parameters	Value	Units	Reference ranges
White blood cell count	0.22	10^9^/L	3.50–9.50
Absolute neutrophil count	0.01	10^9^/L	
Red blood cell count	2.13	10^12^/L	4.30–5.80
Platelet count	21	10^9^/L	125–350
Hemoglobin	64	g/L	130–175
Reticulocyte count	0.4	10^9^/L	24.0–84.0
Alanine transaminase	129.0	IU/L	0.0–50.0
Aspartate transaminase	43.0	IU/L	17.0–59.0
Gamma-glutamyl transferase	155.0	IU/L	15.0–73.0
Alkaline phosphatase	90.8	IU/L	38.0–126.0
Total bilirubin	204.9	μmol/L	3.0–22.0

**FIGURE 1:**
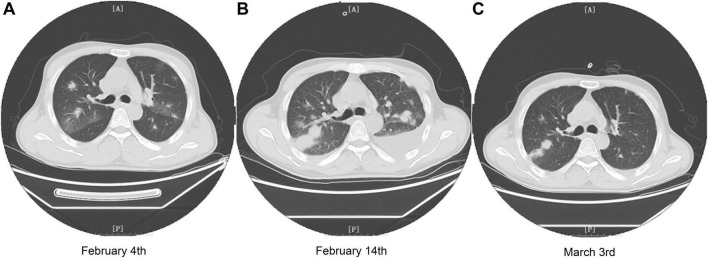
Chest CT images of the patient during treatment in our hospital. **(A)** Chest CT image of the patient on February 4th (2 days before VV116 initiation). **(B)** Chest CT image of the patient on February 14th, 4 days after completing the VV116 course. **(C)** Chest CT image of the patient on March 3rd, 3 days before leaving our hospital.

**FIGURE 2:**
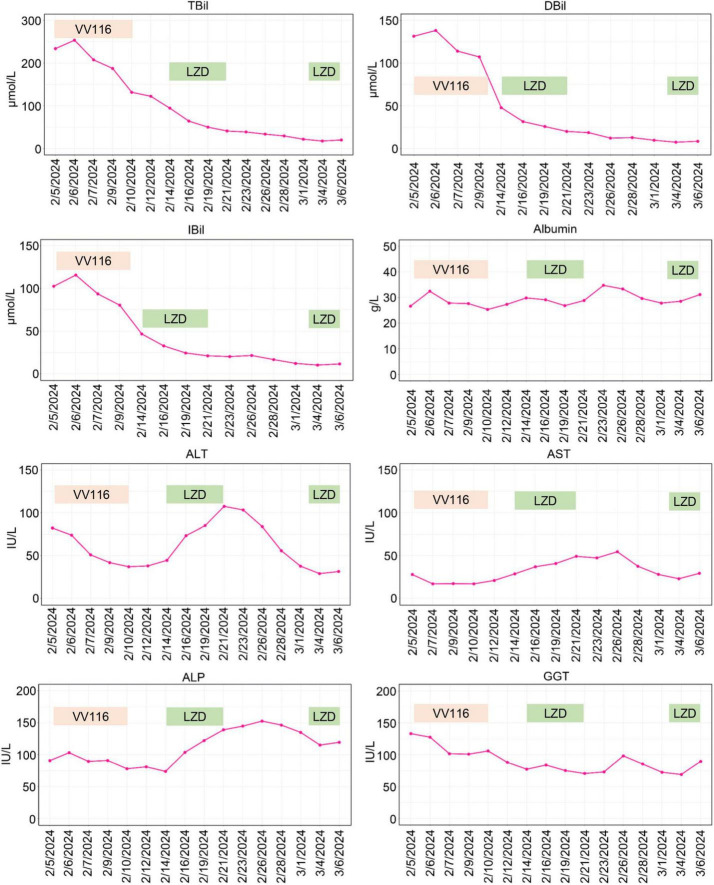
Graphical representation of hepatic enzymes and bilirubin during the patient’s stay in our hospital. TBil, total bilirubin; DBil, direct bilirubin; IBil, indirect bilirubin; ALT, alanine aminotransferase; AST, aspartate aminotransferase; ALP, alkaline phosphatase; GGT, gamma-glutamyl transpeptidase; LZD, linezolid.

**FIGURE 3:**
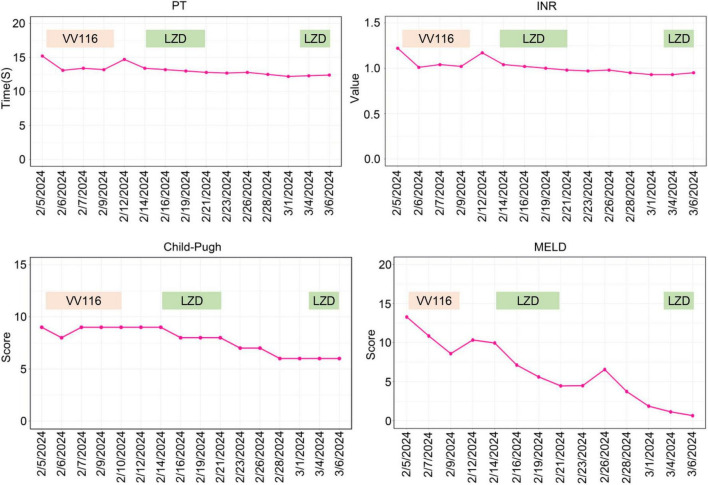
Trends of parameters of coagulation function and scores of Child-Pugh and MELD. PT, prothrombin time; INR, international normalized ratio; LZD, linezolid.

On February 4, 2024, the patient developed a fever with temperature of 38.4°C. Routine blood examination showed C-Reactive Protein of 119.77 mg/L, Serum Amyloid A of 148.83 mg/L. On February 5, 2024, the NGS detection of sputum culture indicated the presence of Epstein-Barr virus, herpes simplex virus and SARS-CoV-2 (Omicron XBB.1). Notably, SARS-CoV-2 exhibited a high sequence number of 52839. Chest CT showed ground glass opacities of both lungs, bilateral mild pleural effusions with atelectasis of right lower lobe, and multiple small nodules (Figure 1A). For antiviral therapy against SARS-CoV-2, we consulted previous reports regarding the clinical use of VV116 and determined the schedule as 300 mg every 12 h on day 1 followed by 200 mg every 12 h on days 2–5 for this patient with severe liver dysfunction and aplastic anemia. The patient and his family members all agreed to this regimen and provided written informed consent. Moreover, broad-spectrum antibacterial medications including meropenem and levofloxacin were initiated to prevent secondary infection. On February 14, 2024, 4 days after completing the VV116 course, NGS of sputum confirmed that SARS-CoV-2 was undetected, indicating successful viral clearance. In the process of VV116 treatment from February 6, 2024 to February 10, 2024, the liver function, coagulation function and kidney function were closely monitored. As shown in Figure 2, the TBil, DBil, IBil, ALT, AST, ALP and GGT levels were declined stepwise; and serum albumin levels remained stable throughout the treatment. Meanwhile, coagulation function markers prothrombin time (PT) and international normalized ratio (INR) were steady within the normal range (Figure 3). Furthermore, to comprehensively evaluate the liver function, we calculated the Child-Pugh score and Model for End-Stage Liver Disease (MELD) score according to corresponding formula (18, 19). As shown in Figure 3, the Child-Push score was stable, and the MELD score was falling steadily during VV116 treatment. The kidney function markers (serum creatinine, blood urea nitrogen, uric acid) also showed no obvious changes during the treatment course of VV116 (Supplementary Figure 1).

However, the pulmonary infection was aggravated on February 14 (Figure 1B). Serum galactomannan testing and sputum culture indicated the existence of *Candia tropicalis*, *Candida parapsilosis* and *Aspergillus terreus complex*. Caspofungin (50 mg, QD) was administered for antifungal therapy. Besides, linezolid (600 mg, QD) was prescribed to cover suspected gram-positive bacterial infections.

Whilst, the levels of ALT, AST and ALP showed mild increase from February 14 to February 21 (Figure 2). Notably, in this period, except hepatoprotective drugs and antibiotics (meropenem and levofloxacin), caspofungin and linezolid were newly prescribed drugs, which might be the etiological agents responsible for transaminase elevations. To evaluate the hepatotoxicity of linezolid and caspofungin, we consulted the LiverTox Web site,^1^ an excellent resource for drug-induced liver injury. Based on LiverTox records, linezolid and caspofungin are both potentially hepatoxic, but linezolid got higher likelihood score of A (Well known cause) compared to caspofungin with a likelihood score of D (Possible cause). Therefore, linezolid was discontinued and replaced by vancomycin (500 mg, Q12H) on February 22, 2024. Then the levels of ALT, AST and ALP were gradually declined (Figure 2). Given the liver function improved significantly on March 3, 2024 and linezolid is superior to vancomycin for the treatment of pneumonia (20), we reintroduced linezolid to replace vancomycin. Notably, the levels of ALT, AST and ALP were back up again on March 6, 2024 as shown in Figure 2, further supporting the earlier elevations of ALT, AST and ALP from February 14 to February 21 might be ascribed to the application of linezolid. In contrast to linezolid treatment, VV116 administration (from February 6, 2024 to February 10, 2024) did not induce any elevation of liver enzymes or bilirubin (Figure 2), demonstrating the good tolerance of VV116 in patients with liver impairment.

On March 3, 2024, NGS detection of sputum demonstrated the sequence number of *Candia tropicalis* and *Aspergillus terreus complex* were significantly declined, and CT scan also confirmed the pulmonary function improved significantly (Figure 1C). By March 6, 2024, fever and cough with expectoration had no happened to the patient for several days. Jaundice nearly disappeared. Considering the improved general conditions and the insufficiency of blood supply in our hospital, the patient was discharged from our hospital and transferred to local hospital for blood transfusion and treatment of aplastic anemia.

## 3 Discussion

To ascertain the appropriate dosage of VV116 for COVID-19 patients with severe liver dysfunction, we consulted previous reports on the clinical use of VV116. Firstly, phase I clinical trials revealed no serious adverse events happened in healthy subjects receiving VV116 at doses of 200–600 mg Q12H, with only one subject receiving 400 mg VV116 experienced a mild and transient transaminase increase (21). Secondly, an open, prospective cohort study showed 7 out of 60 COVID-19 patients taking VV116 (300 mg, Q12H for 5 days) had mild liver enzyme elevations, which resolved spontaneously (22). Thirdly, pharmacokinetic analysis indicated a dosage of 200 mg Q12H could achieve effective concentrations against SARS-CoV-2 (21, 23, 24).

VV116 was developed from remdesivir. Remdesivir exhibited no hepatotoxicity in preclinical study. However, in clinical trials, remdesivir might induce transient elevations of aminotransferases (25). In contrast, VV116, which has a wide tissue distribution, exhibits lower liver-targeting capability and enhanced lung-specific delivery (26). Our study indicated VV116 was well-tolerated in COVID-19 patients with liver impairment. Notably, our main concern regarding this conclusion is whether the good tolerance of VV116 can be ascribed to the concomitant use of hepatoprotective drugs. However, as aforementioned, during the treatment course from February 14 to February 21, linezolid induced an elevation of transaminases even under hepatoprotective treatment. Previous studies have also demonstrated linezolid could lead to liver impairment (27). Generally, linezolid-induced liver injury occurs under conditions of prolonged or high-dose administration (27, 28). In this study, a standard dose of linezolid treatment for only a few days induced an elevation of transaminases, indicating our patient was highly sensitive to hepatotoxic drugs even under hepatoprotective therapy. In contrast, during the VV116 treatment for COVID-19, the liver function of the patient continued to improve gradually, supporting the favorable tolerance of VV116 in COVID-19 patients with liver dysfunction.

## 4 Conclusion

Our study indicated VV116 was well-tolerated in COVID-19 patients with liver impairment. Notably, our main concern regarding this conclusion is whether the blood concentration of VV116 in patients with liver impairment differs from that in the general population. A limitation of this study is the lack of monitoring of blood drug concentrations. Future studies involving similar patients may consider blood drug concentration monitoring or conducting population pharmacokinetic studies.

## Data Availability

The original contributions presented in this study are included in this article/Supplementary material, further inquiries can be directed to the corresponding authors.

## Footnotes

1
https://www.ncbi.nlm.nih.gov/books/NBK547852/
