# Supplementary material for: Dose determination of VV116 in COVID-19 patients with severe liver dysfunction: a case report
Source: Front Med (Lausanne). 2025 Feb 25;12:1541235. doi: 10.3389/fmed.2025.1541235 (PMC11893389; doi:10.3389/fmed.2025.1541235)
Supplement: Supplementary file 1 [file Supplementary_file_1.docx]

***Supplementary Material***

**Dose determination of VV116 in COVID-19 patients with severe liver dysfunction: a case report**


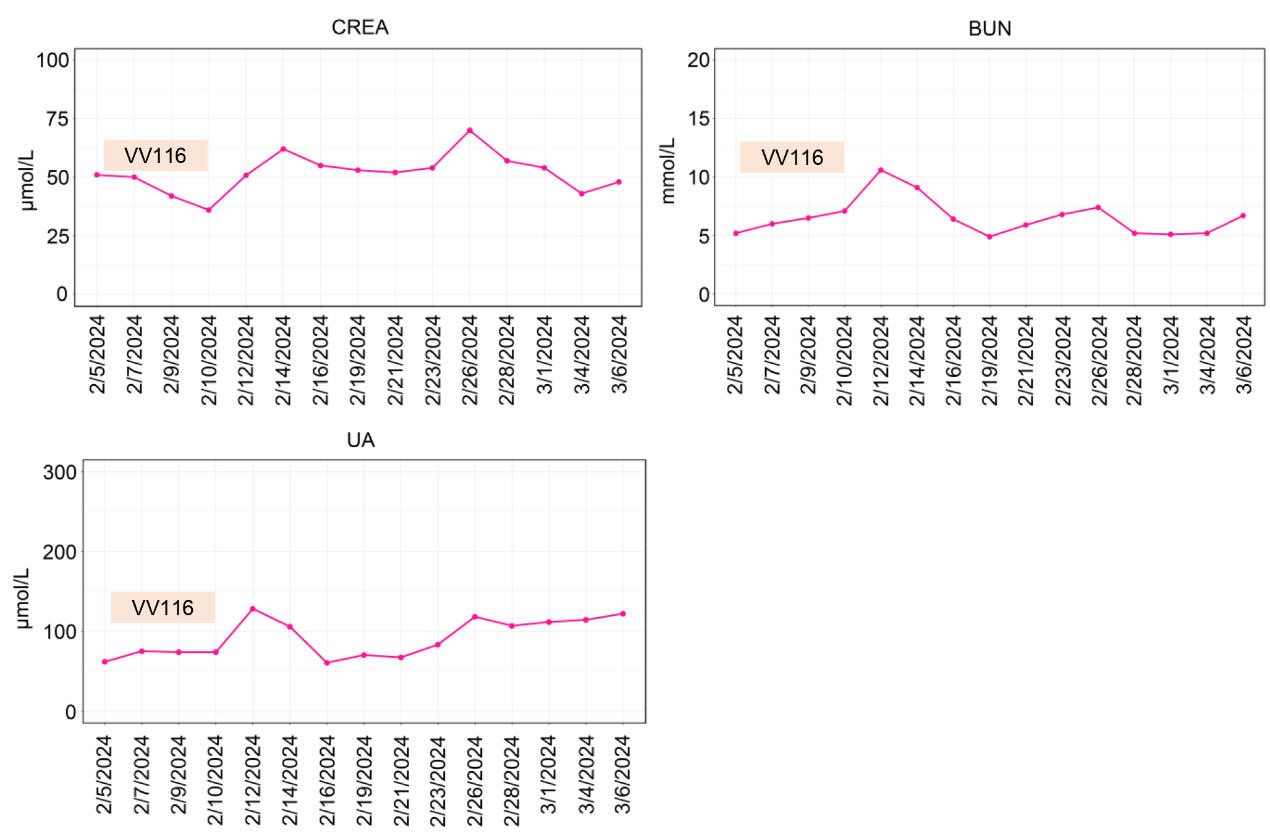


**FIGURE S1 Changes of kidney function markers of the patient during treatment in our hospital**. CREA: Creatinine (normal range: 58.0-110.0 μmol/L); BUN: Blood urea nitrogen (normal range: 3.2-8.0 mmol/L); UA: Uric acid (normal range: 208.0-506.0 μmol/L)
